# Demographic and socio-economic correlates of knowledge of the ovulatory cycle among tribal women in India: Evidence from the nationally representative survey (NFHS-5)

**DOI:** 10.1186/s12889-024-18296-1

**Published:** 2024-03-12

**Authors:** Sameer Kumar Jena, Mriganka Dolui, Sucharita Ghoshal, Sanjit Sarkar

**Affiliations:** 1 Department of Population Studies, Fakir Mohan University, Balesore, Odisha 756089 India; 2 Department of Geography, Central University of Karnataka, Kalaburagi, Karnataka 585311 India; 3 Department of Agriculture, Rural and Tribal Development, Ramakrishna Mission Vivekananda Educational and Research Institute, Morabadi, Ranchi, Jharkhand 834008 India

**Keywords:** India, Tribal communities, Reproductive health, Ovulatory cycle, Knowledge

## Abstract

**Background:**

The knowledge of ovulatory cycle (KOC) is the basis for natural family planning methods. The absence of knowledge is a notable issue since the ovulatory cycle plays a crucial role in reproductive health and empowers women to make informed decisions that influence their lives. This study examines the knowledge of the ovulatory cycle among reproductive tribal women in India and its demographic and socio-economic determinants.

**Methods:**

The data were derived from the National Family Health Survey conducted in 2019–2021. The effective sample size for the present study was 1,01,914 tribal women aged 15–49 years in India. Descriptive statistics along with bivariate analysis were conducted to find the preliminary results. Additionally, multivariable binary logistic regressions were conducted to determine the likelihood of KOC among tribal women across different characteristics. We conducted statistical analysis in STATA 17.0 (StataCorp) and used ArcGIS 10.8.2 for spatial mapping.

**Results:**

Out of 1,01,914 tribal women, 78.8 per cent lack correct knowledge of the ovulatory cycle. Notably, Education level significantly influences KOC, with secondary education showing higher odds of KOC (AOR: 1.24, 95% CI:1.006–1.528) compared to no education. Christian women exhibit lower odds of having KOC (AOR: 0.749, 95% CI:0.564–0.996) compared to Hindu women. Husband/partner’s education level shows a strong association, with higher-educated partners correlating with higher odds of KOC (AOR: 2.501, 95% CI: 1.807–3.461) for higher education. Knowledge of any contraceptive method and current contraceptive use type are strongly associated with KOC. Additionally, rural residence negatively influences KOC (AOR: 1.545, 95% CI: 1.236–1.932), while exposure to mass media has a positive effect (AOR: 1.152, 95% CI: 0.975–1.362) albeit modest.

**Conclusion:**

The study highlights the need for targeted educational and awareness programs to improve KOC among tribal women in India. By addressing factors such as education, religious influences, and place of residence, we can empower these women to make informed decisions about their reproductive health, ultimately enhancing their overall well-being and quality of life. This knowledge is not only a foundation for natural family planning but also a key driver of women’s agency and autonomy in shaping their lives.

## Introduction

The tribal communities of India numbering in the millions are an integral part of the nation’s social fabric, contributing to its cultural diversity and heritage. Nevertheless, despite their unique customs and ways of life, tribal women continue to face a harsh reality of being overlooked, disadvantaged, and generally uninformed about crucial elements of their reproductive health, such as the ovulatory cycle, contraception, and pregnancy [1, 2]. The ovulatory cycle, a fundamental aspect of fertility and reproductive health, continues to puzzle many individuals, significantly impacting their lives. Comprehending the reproductive cycle is considered essential for using any family planning strategies (FPS). These help to determine the fertile window for sexual activity, utilizing either physical indicators or temporal changes [3–6].

Understanding the ovulatory cycle is essential for women everywhere, as it forms the foundation of reproductive health and family planning. This monthly process, characterized by a complex interplay of hormones and physiological changes, culminates in the release of an egg for potential fertilization [6–8]. Knowledge of this cycle empowers women to make informed decisions about family planning, contraception methods, and overall reproductive health—choices that profoundly impact the trajectory of their lives [5, 7]. The lack of knowledge about the ovulatory cycle, contraception methods, and pregnancy poses profound implications for their well-being, family planning, and overall quality of life. Tragically, for most tribal women across India, this critical information remains uncharted territory, perpetuating a cycle of vulnerability and disempowerment [3–5, 7, 8].

There are many reasons behind this knowledge gap among Indian Scheduled tribe (ST) that are deeply intertwined with historical, cultural, and systemic factors. Tribal societies often maintain oral traditions, passing down knowledge through storytelling and customs, which can lead to gaps in essential information, especially in health matters [9–11]. The absence of health awareness programs and healthcare facilities in remote tribal areas leaves tribal women in the dark about their bodies [12–14] Cultural taboos regarding reproductive health and menstruation exacerbate the problem. In many tribal communities, menstruation is steeped through the lens of stigma and secrecy, causing women to conceal their experiences and questions about the ovulatory cycle [15–17]. Moreover, socioeconomic disparities exacerbate the issue, with limited access to education and economic opportunities leaving many tribal communities trapped in a cycle of poverty [18]. It hinders their ability to seek information or make informed choices regarding their reproductive health. Geographical isolation often results in inadequate healthcare infrastructure, making it difficult for tribal women to access reliable sources of information about the ovulatory cycle, contraception, and pregnancy [14, 19, 20]. The consequences of this pervasive lack of knowledge are far-reaching and profound. Unintended pregnancies, maternal health issues, and a higher risk of sexually transmitted infections are just some of the hardships faced by tribal women who remain uninformed about their reproductive health [4, 8]. These challenges affect individual women and have broader implications for their communities’ well-being and future generations’ prospects [3, 7].

Empowering women in Indian tribal communities with knowledge about the ovulatory cycle is not just a matter of addressing their immediate health concerns; it is a pivotal step towards fostering gender equality and advancing social progress within these communities. When women have the tools to make informed decisions about their reproductive health, it enhances autonomy over their lives and futures. Education about the ovulatory cycle can serve as a catalyst for improved family planning, healthier pregnancies, and the development of more robust and resilient tribal communities. The knowledge of the ovulatory cycle (KOC) is the base for natural family planning methods [21]. Natural family planning (NFP), introduced in 1970 and distinguished from contraceptives, is part of family planning methods along with contraceptives and fertility awareness-based methods (FABM). This approach empowers women to view fertility as a normal biological process, allowing them to take control of their reproductive health [22]. Within NFP, couples learn to plan or delay pregnancy by closely observing physiological signs such as cervical mucus, basal body temperature (BBT), and measuring urinary hormones. These indicators help determine the woman’s fertility status throughout her menstrual cycle. If a couple desires pregnancy, they focus on maximizing the potentially fertile phase. Conversely, if they wish to avoid or delay pregnancy, abstinence from sexual intercourse is recommended during the fertile window. Fertility awareness-based methods (FABMs) of family planning rely on physical signs and symptoms that change with hormone fluctuations during a woman’s menstrual cycle to predict her fertility [22, 23].

In the contemporary landscape of public health, where increased health risks, denials, terminations, and a substantial need for modern methods of contraception, it becomes imperative to examine the knowledge of the ovulatory period among reproductive tribal women in India [21]. This understanding is not merely a matter of academic interest but forms the bedrock for recommending Natural Methods of Family Planning (NMFPs) as a viable alternative to modern contraceptives [4, 24]. By identifying and understanding the factors related to ovulatory cycle knowledge, reproductive tribal women may utilize NMFPs more frequently than modern contraceptives [4]. This shift could address the current challenges faced in contraceptive use and pave the way for improved reproductive health outcomes [24]. Tribal women in India are more vulnerable in terms the knowledge of their menstrual cycle and use of hygienic period products [25]. Hence, their choice to consult informal health practitioners for abortion care services stemmed from a lack of information about preventing unwanted pregnancies and uncertainty about where to access high quality [26]. Moreover, there is a discernible distinction in the use of period products during menstruation between tribal women and non-tribal women in India due to economic conditions, educational level and lack of knowledge [27].

However, a significant gap exists in the existing literature. Very few studies have addressed the association of socio-economic factors with the knowledge of the ovulatory cycle (KOC) among indigenous reproductive women [4, 21, 28, 29]. This gap is particularly concerning given the potential implications of KOC on contraceptive use and reproductive health outcomes.

Therefore, this present study aims to fill this gap by focusing on the knowledge of the ovulatory cycle (KOC) and its demographic and socioeconomic determinants among reproductive tribal women in India. Additionally, state-level clustering and district-level spatial distribution are incorporated to examine the regional variation in Knowledge of the Ovulatory Cycle (KOC) among indigenous reproductive-aged women in India. This may aid policymakers in formulating specific policies and programs for the targeted group. Moreover, it could enhance women’s awareness by fostering a proper understanding of Knowledge of the Ovulatory Cycle (KOC).

## Data and methods

### Data source

The most recent inclusive data available from the National Family Health Survey, round 5 (NFHS-5; 2019–2021) conducted by the International Institute for Population Sciences (IIPS) under the supervision of the Ministry of Health and Family Welfare (MoHFW) of the Indian Government, holds significant potential in dissecting and elucidating the aforementioned domains and their interconnections [30]. The survey encompassed a large sample size, including 6,36,699 households, 1,01,839 men aged 15–54, and 7,24,115 eligible women aged 15–49. The NFHS-5 (2019–2021) presents extensive data covering a wide range of areas, encompassing population demographics, child and maternal health, family planning, nutritional indicators, as well as adult health, for India, each state/union territory (UT), and 707 districts as of March 31, 2017.

Therefore, this study was based on the recent release of the National Family Health Survey (NFHS-5), to fulfil the objective of this study. The findings of our study is based on a total number of 1,01,914 Scheduled Tribe (ST) women aged 15–49 years from NFHS-5. The detailed information regarding the data capturing methodology, including the sampling frame, survey design, and data collection process, is mentioned in a publication elsewhere [30].

## Variable description

### Dependent variable

The analysis consists of Knowledge of the Ovulatory Cycle (KOC) as a dependent variable, based on the existing responses from women. Women were asked *“When do you think a woman has the greatest chance of becoming pregnant?”* and answers were recorded as “during her period”, “after the period ended”, “middle of the cycle”, “before the period begins”, “at any time”, “other” and “don’t know”. For our study, we categorized women into two groups according to KOC. Depending on the women’s responses, they were classified as ‘having accurate KOC’ who answered - were at “the middle of the menstrual cycle” and ‘not having accurate KOC’ refers to those who responded the fertile period was “during her period”, “after the period ended”, “before period begins”, “at any time”, “others” and “don’t know” [21].

### Independent variable

In this study, a set of explanatory variables was used to explore the determinants of KOC. The variables are Age (years), Education, Religion, Marital Status, Wealth Index, Number of living children, Husband or partner’s education level, Region, Residence, Knowledge of any contraceptive method, Current use by method type, Exposure to Mass Media, and Digital Literacy. Table 1 contains the explanation of the description of these variables.

**Table 1:** Descriptions of independent variables

Independent variables	Descriptions	Coding
Age (years)	Women were asked about their current age. For the analysis, age has been classified into three categories: 15–24 years old, 25–34 years old and 35–49 years old.	1 = 15–24 years, 2 = 25–34 years and 3 = 35–49 years
Education	Women were asked about the number of years they had spent in school. We considered this indicator as level of education which is classified into four categories: No education, Primary, Secondary, and Higher	1 = No education, 2 = Primary, 3 = Secondary and 4 = Higher
Religion	Women were asked about their religion which was recorded as Hindu, Muslim, Christian, Sikh, Buddhist/Neo Buddhist, Jain, Jew, Parsi/Zoroastrian, Others, and no religion. We classified the religious category into three categories: Hindu, Muslim, Christian and Others	1 = Hindu, 2 = Muslim 3 = Christian and 4 = Others
Marital Status	In the NFHS-5, women were asked about their marital status which was recorded as “never in union”, “married”, “living with a partner”, “widowed”, “divorced”, or “no longer living together/separated”. We have classified marital status is classified into three categories: Unmarried (if the woman had never been in a union), Married (if the woman married and living with her partner), and Others (if the woman widowed, divorced no longer living together/separated).	1 = Unmarried, 2 = Married and 3 = Others.
Wealth index	NFHS-5 assessed household wealth using wealth scores derived from principal component analysis, considering indicators such as consumer goods ownership and housing features. Following this, households were divided into five equal groups, known as quintiles or wealth quintiles. The wealth quintiles were further categorized into 20% each, including the Poorest, Poorer, Middle, Richer, and Richest. We have used these indicators as provided within the datasets.	1 = Poorest, 2 = Poorer, 3 = Middle, 4 = Richer and 5 = Richest
Region	NFHS-5 provided the states category and we have classified the states into geographical regions based on direction. The geographical region is classified into six regions: North, Central, East, Northeast, West and South.	1 = North, 2 = Central, 3 = East, 4 = Northeast, 5 = West and 6 = South.
Residence	The place of residence of women is recorded as Urban and Rural. Thus, we have considered these categories as places of residence: Urban and Rural	1 = Urban and 2 = Rural
Number of living children	Women were asked about the number of children who are alive which is classified into three: No children, 1–2 and 3 or more than 3.	0 = No children, 1 = 1–2 and 2 = 3 or more
Husband/partner’s education level	Women were asked about their husband/partners’ educational status, specifically the number of years they had spent in school. We considered this indicator as a level of education, which was classified into four categories: No education, Primary, Secondary, and Higher.	0 = No education, 1 = Primary, 2 = Secondary, and 3 = Higher
Knowledge of any contraceptive method	Women were asked about contraceptive methods by “knowledge of any method” and options were “knows no method”, “knows only folkloric method”, “knows only traditional method” and “knows modern method”. We have categorised into three: No method, Traditional method folkloric method and traditional), Modern method	0 = No method, 1 = Traditional method, and 2 = Modern method
Current use by method type	In NFHS-5 women were asked about “current use by method type” and responses were recorded as “no method”, “folkloric method”, “traditional method”, and “modern method”. We have classified into three categories: No method, Traditional method (folkloric method and traditional), Modern method	0 = No method, 1 = Traditional method, and 2 = Modern method
Exposure to Mass Media	Women were asked about their exposure to different mass media such as “frequency of reading newspaper or magazine”, “frequency of listening to radio”, and “frequency of reading newspaper or magazine” and options were “not at all”, “less than once a week” and “at least once a week”. We have used these variables as exposure to media and classified them in “No” and “Yes”.	0 = Not Exposed and 1 = Exposed
Digital Literacy	NFHS-5 recorded several information that may derived the knowledge of digital accessibility such as “owns a mobile telephone”, “use a mobile telephone for financial transactions”, “have you ever used the internet?”, “are you able to read text (SMS) messages?” and responses are coded as “No” and “Yes”. Further, we have used these variables as digital literacy and categorised as no and yes.	0 = No and 1 = Yes

## Statistical analysis

We used bivariate statistics to analyse the weighted prevalence of KOC among women belonging to tribal category across various background characteristics. Furthermore, we assessed the statistical performance for the significance of the association between each independent variable and the dependent variables using a chi-square (*χ²*) test. Thereafter, a multivariable binary logistic regression model has been used to show the determinants and likelihood of KOC. We reported adjusted odds ratio (AOR), and p-values at 95% confidence intervals (95% CI) to represent the outcome of the logistic regression model. We have calculated the variance inflation factors (VIF) to test multicollinearity issues among independent variables and results show no collinearity exists among the independent variables (Maximum VIF = 2.06, and Minimum VIF = 1.02). The Akaike information criterion (AIC) and log-likelihood were used as an overall model fitting statistic in the logistic regression model. Besides, a spatial mapping tool was used to show the geospatial patterning of KOC in all districts. All analyses stood weighted (V005/10,00,000) to get unbiased estimates. All statistical analyses were conducted using STATA 17.0 (StataCorp), and for spatial mapping, ArcGIS (10.8.2) software was employed.

## Results

Table 2 presents a detailed overview of the demographic and socio-economic characteristics of tribal women in India. The tribal women population distributed across various age groups in India, and the most significant proportion falls within the 35–49 age range, accounting for 35.9%. Regarding education, more than half have received a secondary education (52.2%), while a substantial portion lacks formal education (25.01%). In terms of religion, Hinduism is practices by the majority faith (46.6%), followed by Christianity (39.8%). The wealth index shows that a significant proportion of these women belong to the “poorest” category (36.6%). Most of them are married (66.9%) and have at least one or two living children (35.2%). Their partners also have varying education levels, with a substantial percentage having received a secondary education (50.2%). Notably, an overwhelming majority of these women are knowledgeable about modern contraceptive methods (98.4%), and a significant percentage currently use these methods (33.2%). Geographically, the Northeastern region has the highest representation (53.6%), and rural areas are predominant (84.8%). Digital literacy remains low, with the majority lacking digital skills (89.2%).

**Table 2:** Demographic and Socio-economic characteristics of Tribal women in India

Background Characteristics	Percentage		N
Age			
15–24	32.48		33,099
25–34	31.57		32,178
35–49	35.95		36,637
Education			
No education	25.01		25,485
Primary	13.40		13,656
Secondary	52.20		53,203
Higher	9.39		9570
Religion			
Hindu	46.61		47,504
Muslim	2.98		3033
Christian	39.80		40,562
Others	10.61		10,815
Marital status			
Unmarried	27.96		28,491
Married	66.96		68,244
Wealth index			
Poorest	36.64		37,338
Poorer	28.03		28,569
Middle	18.53		18,888
Richer	11.65		11,871
Richest	5.15		5248
Region			
North	7.28		7423
Central	9.14		9318
East	17.94		18,285
Northeast	53.62		54,651
West	7.23		7364
South	4.78		4873
Residence			
Urban	15.20		15,491
Rural	84.80		86,423
Number of living children			
No children	34.43		35,086
1–2	35.28		35,951
3 or more	30.30		30,877
Husband/partner’s education level			
No education	23.97		2683
Primary	16.36		1831
Secondary	50.23		5623
Higher	9.45		1058
***Total***			***11,195***
Knowledge of any contraceptive method			
No method	1.46		1489
Traditional method	0.13		134
Modern method	98.41		100,291
Current use by method type			
No method	59.26		60,396
Traditional method	7.45		7595
Modern method	33.29		33,923
Exposure to Mass Media			
Not Exposed	31.05		31,640
Exposed	68.95		70,274
Digital Literacy			
No	89.27		90,974
Yes	10.73		10,940
Total	100.00		101,914

Table 3 shows valuable insights into the bivariate association between knowledge of the ovulatory cycle (KOC) and various socio-demographic characteristics among tribal women in India. The data reveals statistically significant associations between KOC and several factors. Notably, there is a clear relationship between age and KOC, with the highest percentage of knowledge found among women in the age group 25–34 (23.6%) and 35–49 (23.4%). Education level also plays a significant role, as those with higher education levels tend to have better KOC, 26.9 per cent of tribal women with Higher education have KOC. Similarly, religion, wealth index, marital status, and the number of living children show significant correlations with KOC. Additionally, women whose husbands or partners have higher education levels exhibit a higher level of KOC, with the highest percentage in the “Higher” education category (33.9%). Women with Knowledge of modern contraceptive methods (21.52%) who lived in western (27.68%) and southern (26.84%) regions, urban as a place of residence (24.48%), and exposure to mass media (23%) also demonstrate strong associations with KOC, with varying percentages. Interestingly, while digital literacy showed a statistically significant association, its impact appears relatively modest compared to other factors. These findings underscore the importance of considering various demographic and socioeconomic factors when addressing and promoting knowledge of the ovulatory cycle among tribal women.

**Table 3:** bivariable association between KOC and demographic and socioeconomic characteristics

Background Characteristics	Knowledge of ovulatory cycle (KOC) %	N	χ²- test
			*P*-value
Age			0.000
15–24	16.73	33,099	
25–34	23.67	32,178	
35–49	23.47	36,637	
Education			0.000
No education	18.74	25,485	
Primary	20.95	13,656	
Secondary	22.02	53,203	
Higher	26.92	9570	
Religion			0.000
Hindu	21.53	47,504	
Muslim	20.37	3033	
Christian	19.52	40,562	
Others	20.63	10,815	
Marital status			0.000
Unmarried	13.13	28,491	
Married	24.02	68,244	
Others	20.71	5179	
Wealth index			0.000
Poorest	17.79	37,338	
Poorer	21.30	28,569	
Middle	24.63	18,888	
Richer	26.69	11,871	
Richest	26.20	5248	
Region			0.000
North	16.64	7423	
Central	19.66	9318	
East	14.94	18,285	
Northeast	25.45	54,651	
West	27.68	7364	
South	26.84	4873	
Residence			0.000
Urban	24.48	15,491	
Rural	20.64	86,423	
Number of living children			0.000
No children	15.43	35,086	
1–2	25.12	35,951	
3 or more	22.25	30,877	
Husband/partner’s education level			0.000
No education	17.07	2683	
Primary	20.29	1831	
Secondary	27.16	5623	
Higher	33.92	1058	
***Total***	***23.83***	***11,195***	
Knowledge of any contraceptive method			0.000
No method	2.17	1489	
Traditional method	14.07	134	
Modern method	21.52	100,291	
Current use by method type			0.000
No method	17.51	60,396	
Traditional method	26.08	7595	
Modern method	25.16	33,923	
Exposure to Mass Media			0.000
Not Exposed	17.79	31,640	
Exposed	23.00	70,274	
Digital Literacy			0.004
No	21.13	90,974	
Yes	22.32	10,940	
Total	21.23	101,914	

Table 4 presents the results of a multivariable binary logistic regression analysis, exploring the association between knowledge of the ovulatory cycle (KOC) and various socio-economic characteristics. Education level significantly influences KOC, with secondary education showing higher odds of KOC (AOR: 1.24, 95% CI:1.006–1.528) compared to no education. Religion also plays a vital role, with Christian women less likely to have KOC (AOR: 0.749, 95% CI: 0.564–0.996) compared to Hindu women. The wealth index exhibits varying associations with no apparent pattern. Marital status significantly influences KOC, with both married (AOR: 6.70, 95% CI:0.33-138.29) and “others” (AOR: 7.25, 95% CI: 0.35–150.80) categories showing higher odds compared to unmarried women. The number of living children does not appear to influence KOC strongly. Husband/partner’s education level shows a strong association, with higher-educated partners correlating with higher odds of KOC (AOR: 2.501, 95% CI: 1.807–3.461) for higher education. Knowledge of any contraceptive method and current contraceptive use type are strongly associated with KOC. Women using traditional methods have significantly higher odds (AOR: 82.999, 95% CI: 1.29-5348.66) of KOC, as do those using modern methods (AOR: 33.7, 95% CI: 0.916-1239.69). The region also plays a significant role, with the Northeastern (AOR: 1.782, 95% CI: 1.308–2.428) and Southern (AOR: 1.634, 95% CI: 1.225–2.179) regions having higher odds of KOC compared to the Northern region. Rural residence negatively influences KOC (AOR: 1.545, 95% CI: 1.236–1.932), while exposure to mass media has a positive effect (AOR: 1.152, 95% CI: 0.975–1.362) albeit modest. Digital literacy does not appear to have a strong association with KOC (AOR: 1.044, 95% CI: 0.88–1.238). The Akaike information criterion (AIC) and log-likelihood statistics fitted the regression model result where the AIC value is 5238.12, and the log-likelihood value is -2587.06 indicating a higher fitted model.

**Table 4:** Multi-variable association (binary logistic regression) between KOC and socio-economic and demographic characteristics

Background Characteristics	KOC				
	Unadjusted OR	*P*-value		Adjusted OR	*P*-value
Age					
15–24®	1			1	
25–34	1.543 [1.455–1.637]	0.000		0.976 [0.789–1.207]	0.823
35–49	1.526 [1.441–1.616]	0.000		1.107 [0.879–1.393]	0.388
Education					
No education®	1			1	
Primary	1.150 [1.065–1.240]	0.000		0.933 [0.745–1.17]	0.550
Secondary	1.225 [1.160–1.293]	0.000		1.24 [1.006–1.528]	0.044**
Higher	1.598 [1.468–1.738]	0.000		0.95 [0.646–1.397]	0.794
Religion					
Hindu®	1			1	
Muslim	0.933 [0.818–1.064]	0.300		1.452 [0.952–2.215]	0.083*
Christian	0.884 [0.819–0.955]	0.002		0.749 [0.564–0.996]	0.047**
Others	0.948 [0.844–1.064]	0.365		0.77 [0.535–1.108]	0.160
Marital status					
Unmarried®	1			1	
Married	2.091 [1.964–2.227]	0.000		6.70 [0.33-138.29]	0.218
Others	1.727 [1.536–1.942]	0.000		7.25 [0.35–150.80]	0.201
Wealth index					
Poorest®	1			1	
Poorer	1.251 [1.178–1.328]	0.000		1.088 [0.906–1.306]	0.366
Middle	1.51 [1.413–1.614]	0.000		0.994 [0.794–1.244]	0.956
Richer	1.682 [1.557–1.817]	0.000		1.034 [0.785–1.361]	0.813
Richest	1.641 [1.501–1.794]	0.000		0.73 [0.498–1.071]	0.107
Region					
North®	1			1	
Central	1.226 [1.115–1.349]	0.000		1.188 [0.914–1.545]	0.198
East	0.88 [0.803–0.966]	0.007		0.793 [0.614–1.025]	0.077*
Northeast	1.711 [1.553–1.885]	0.000		1.782 [1.308–2.428]	0.000***
West	1.918 [1.752-2.1]	0.000		1.503 [1.173–1.926]	0.001***
South	1.839 [1.662–2.034]	0.000		1.634 [1.225–2.179]	0.001***
Residence					
Urban®	1			1	
Rural	0.802 [0.755–0.853]	0.000		1.545 [1.236–1.932]	0.000***
Number of living children					
No children®	1			1	
1–2	1.838 [1.734–1.947]	0.000		1.11 [0.85–1.45]	0.441
3 or more	1.568 [1.473–1.67]	0.000		0.973 [0.72–1.313]	0.856
Husband/partner’s education level					
No education®	1			1	
Primary	1.236 [0.993–1.539]	0.057		1.196 [0.95–1.507]	0.128
Secondary	1.811 [1.527–2.147]	0.000		1.599 [1.305–1.959]	0.000***
Higher	2.493 [1.946–3.193]	0.000		2.501 [1.807–3.461]	0.000***
Knowledge of any contraceptive method					
No method®	1			1	
Traditional method	7.38 [2.53–21.52]	0.000		82.99 [1.29-5348.66]	0.038**
Modern method	12.35 [7.21–21.17]	0.000		33.7 [0.916-1239.69]	0.056*
Current use by method type					
No method®	1			1	
Traditional method	1.662 [1.516–1.821]	0.000		1.303 [1.002–1.694]	0.048**
Modern method	1.584 [1.509–1.662]	0.000		1.293 [1.098–1.524]	0.002***
Exposure to Mass Media					
Not Exposed®	1			1	
Exposed	1.38 [1.312–1.452]	0.000		1.152 [0.975–1.362]	0.097*
Digital Literacy					
No®	1			1	
Yes	1.073 [0.990–1.163]	0.086		1.044 [0.88–1.238]	0.622
***Chi-square (χ²)***	234.977				
***Akaike crit. (AIC)***	5238.123				
***Log likelihood***	-2587.0616				

Note: KOC = Knowledge of the ovulatory cycle; OR- Odds ratio; CI- Confidence Interval at 95% significance level; ®- Reference Category; Level of Significance for the test of association (Pearson’s Chi-Square test); AIC = Akaike’s information criterion

**Fig. 1:**
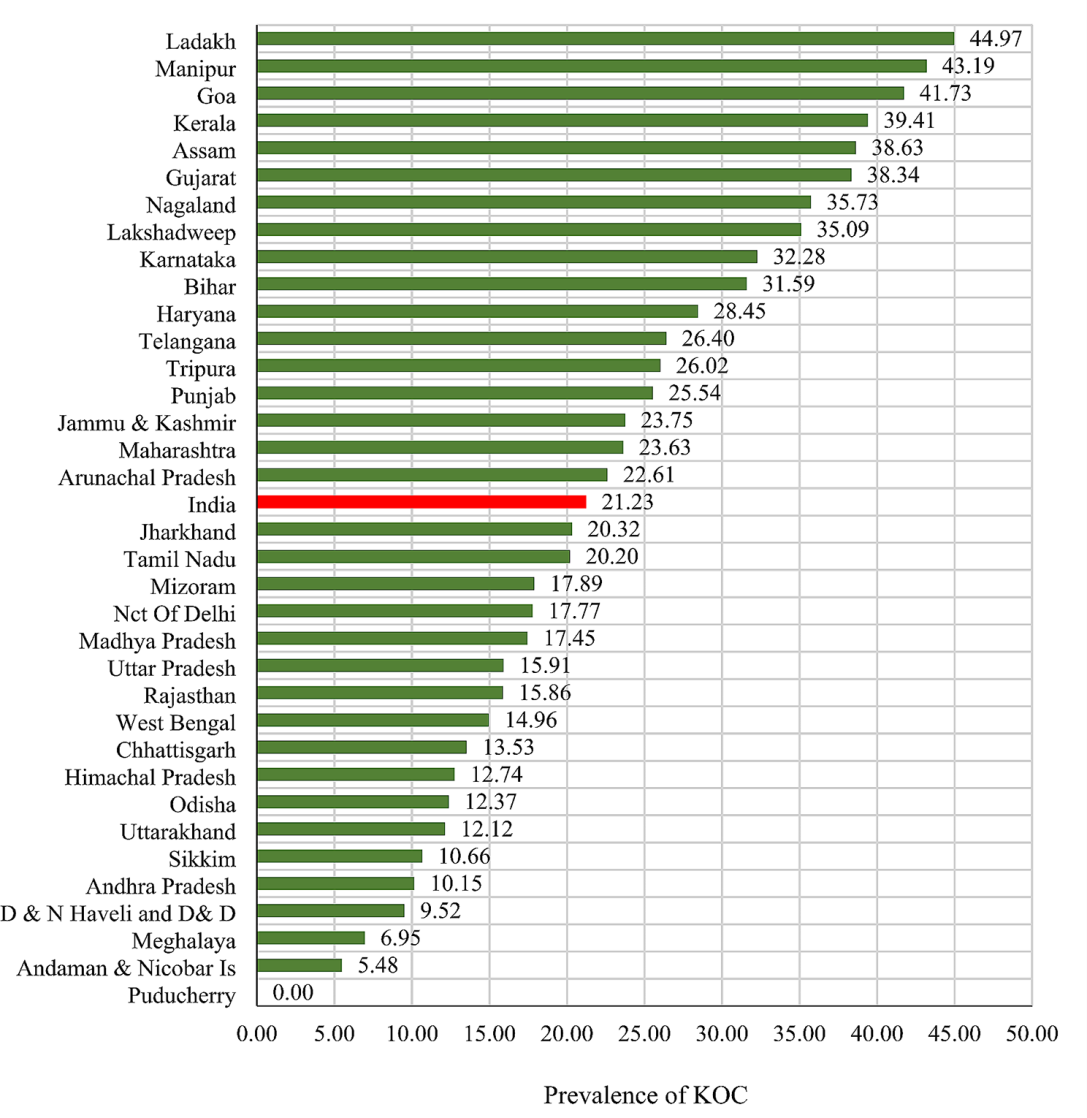
Prevalence of knowledge of the ovulatory cycle (KOC) among tribal women in different states and India

Figure 1 shows the prevalence of knowledge of the ovulatory cycle in various states and union territories in India. It is evident that there is significant variation across the country, with some regions like Puducherry having a very low prevalence, while others like Ladakh and Manipur have higher prevalence rates at 44.97% and 43.19% respectively. Generally, southern states like Kerala, Goa, and Karnataka, along with some northeastern states, tend to have higher levels of knowledge about the ovulatory cycle, while northern and central states like Bihar, Haryana, and Madhya Pradesh have lower levels of awareness. Moreover, the prevalence of KOC was similar to the national average (21.95%) in around half of the states (17 out of 37). This result is essential for understanding the variations in reproductive health education and awareness across different regions of India.

**Fig. 2:**
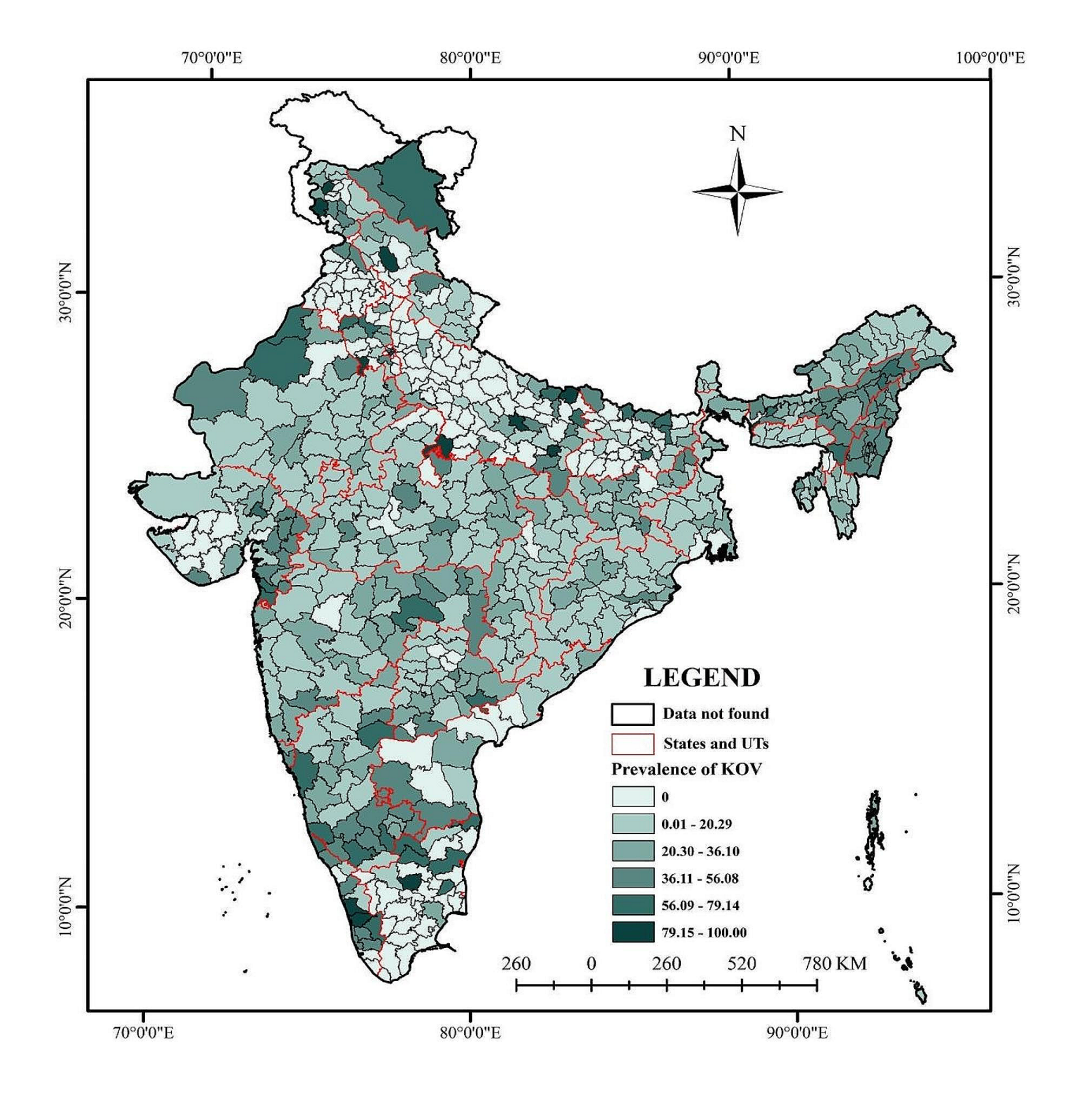
Spatial distribution of the Prevalence of knowledge of the ovulatory cycle (KOC) among women in India (NFHS-5) Note: The shapefile was downloaded from Spatial Data Repository– Boundaries, The Demographic and Health Surveys Program, ICF International, funded by the United States Agency for International Development (USAID). Available from https://spatialdata.dhsprogram.com [Accessed September 01, 2023]. The Spatial Data Repository provides geographically-linked health and demographic data from The DHS Program and the U.S. Census Bureau for mapping in a geographic information system (GIS).

Figure 2 depicts the spatial distribution of KOC of tribal women across districts in India. It highlights significant variations in awareness levels, with some districts, such as Badgam and Majuli, having a 100% knowledge rate, indicating that everyone in these districts is aware of the ovulatory cycle. On the other hand, several districts, including Srinagar, Puducherry, and many in West Bengal, have no knowledge, indicating a complete lack of awareness in these areas. Around 18% of all districts in India (128 out of 707) had a zero prevalence KOC, and a lower level (0.01–20.29%) of KOC was identified in 36% of all the districts (255 out of 707). At the same time, a high level (56.09–79.14%) of KOC is observed among tribal women in 5% of districts (34 out of 707). Additionally, around 2% of districts (14 out of 707) exclusively exhibit full (100%) KOC among tribal women in India. The full KOC districts were majorly located in Uttar Pradesh (Jhansi, Mahrajganj, Varanasi, Amethi), Kerala (Thrissur, Ernakulam), Delhi (East, New Delhi), Himachal Pradesh (Mandi), Haryana (Mahendragarh), Assam (South Salmara Mancachar), Tamilnadu (Namakkal) and Jammu and Kashmir (Badgam, Rajouri). The dataset underscores the need for targeted educational campaigns and interventions to improve reproductive health knowledge in districts with low awareness levels and maintain high awareness in districts where it is already high.

## Discussion

Knowledge of the ovulatory cycle (KOC) is a great concern among Scheduled Tribe women which is a significantly associated factor for maternal health outcomes. Accordingly, our research revealed several notable findings regarding KOC in India.

The findings revealed that the level of education of women was positively and expressively associated factors with KOC. Women with secondary education status were 24% more likely to have KOC (AOR: 1.24, 95% CI:1.006–1.528) compared to those with a primary level of education or no education, these findings align with previous studies [31–34]. Similarly, higher and secondary education level of husbands/partners were 2.5 times (AOR: 2.501, 95% CI:1.807–3.461) and 1.6 times (AOR: 1.599, 95% CI:1.305–1.959) more likely to contributes to women’s KOC respectively than no education. This finding aligns with similar studies, where individuals with higher educational levels are literate and better knowledgeable on aspects associated to reproductive health [35, 36]. Furthermore, when a husband or partner possesses a higher level of education, they tend to be more inclined to engage in discussions about reproductive health. This can potentially contribute to an improved level of women’s KOC [37, 38]. Previous studies in India revealed that mothers do not have proper information regarding KOC and they were reluctant to discuss the problems faced by their daughters [39, 40]. In concern to religion, tribal women who belong to the Christian religion were found to have 25% less likelihood of KOC (AOR: 0.749, 95% CI:0.564–0.996) than compared to Hindus. The possible explanation could stem from the contrast in the religious doctrines of the contemporary contraception methods; thus, they do not depend on and need not know about their fertile period [5, 41]. Nevertheless, our study identified that the prevalence and odds of KOC were higher (26.69%) among tribal women who belong to the richer group as compared to the poorer (17.79%), but our results could not significantly associate with KOC as a determinant factor. However, the wealth index was also significantly associated with women’s KOC in some other studies [38, 7, 42–44].

Likewise, the findings of our study also revealed that tribal women belonging to Northeast, west and southern region of the country were 78% (AOR: 1.782, 95% CI:1.308–2.428), 50% (AOR: 1.503, 95% CI:1.173–1.926) and 63% (AOR: 1.634, 95% CI:1.225–2.179) more knowledgeable of their ovulatory cycles than those women who resides in northern region, respectively. Similarly, districts situated in the northeast, north and southern states exhibited a higher prevalence of KOC. Moreover, findings revealed that tribal women residing in rural areas were 54% more knowledgeable (AOR: 1.545, 95% CI:1.236–1.932) of their ovulatory cycles than those who reside in urban areas. On the contrary, some studies highlighted that women residing in urban areas were more knowledgeable than rural women [7, 45–47]. Another considerable fact was our study primarily focused on tribal women; it is noteworthy that the majority (84.80%) of them reside in rural areas. The reason might be, that had greater access to information regarding reproductive health care and services compared to rural areas from health workers and community health workers [34, 48, 49].

In the study, there was a substantial association between tribal women’s understanding of the Ovulatory Cycle (KOC) and their awareness of any form of contraception. Specifically, Scheduled Tribe women who were knowledgeable about both the Traditional and Modern methods of contraception were 82.99 times and 33.7 times more likely to have KOC. This implies that women with greater exposure to different contraceptive methods tend to be more knowledgeable about the Ovulatory Cycle [50, 51]. Moreover, tribal women who currently used traditional contraception method (AOR: 1.293, 95% CI: 1.098–1.524) and modern contraception methods (AOR: 1.293, 95% CI: 1.098–1.524) were 30% and 29% more likely to have accurate knowledge of their ovulatory cycle compared to those who used no method. There might be a possibility that women who use modern contraceptive methods have proper knowledge of their ovulatory cycle and avoid unintentional pregnancy [48, 49]. It was well-addressed, that KOC was significantly associated with exposure to Mass Media and Digital Literacy. Women who had exposure to Mass Media are 15.2% more likely to have knowledge of the ovulatory cycle (AOR: 1.152, 95% CI: 0.975–1.362) than those who don’t have exposure. In a similar way, women having Digital Literacy had 4% greater odds of KOC (AOR: 1.044, 95% CI: 0.880–1.238) than those who do not have. Corresponding findings were reported in a study [5]. Moreover, mass media and digital literacy were considered as powerful tools or sources to raise awareness and knowledge and influence individual behavioural attitudes toward KOC [35, 52, 53]. This allows to reach a significant information to the rural women of their reproduction health and KOC.

To the best of the authors’ knowledge, this is the first study to assess the strength of KOC and its determinants among ST women of reproductive age in India using the most recent national representative data (NFHS-5). Further, the findings of the research suggested that among tribal women, those with a secondary education level residing in rural areas in the Northeast, West, and Southern regions, as well as having spouses or partners with education levels ranging from secondary to higher, possessing knowledge of various contraceptive methods, and having exposure to mass media, exhibited a significantly higher level of awareness regarding the ovulatory cycle. On the other hand, scheduled tribe women belonging to Christian communities are less aware of the ovulatory cycle.

## Conclusion

The current study provides a comprehensive understanding of the demographic and socio-economic characteristics of tribal women in India and their knowledge of the ovulatory cycle (KOC). Around 21.2% per cent of tribal women have the right Knowledge of the Ovulatory Cycle in India. Thereafter, we found significant associations between KOC and socioeconomic and demographic factors such as education, religion, economic condition (wealth index), region, residence, partner’s education, knowledge of contraceptive methods and use, and exposure to mass media. These findings emphasise the comprehensive nature of factors influencing KOC among tribal women. Geographically, there are substantial variations in awareness levels, with southern and northeastern regions generally exhibiting higher awareness. District-level data reveals significant disparities, highlighting the need for targeted educational campaigns and interventions to enhance reproductive health knowledge in areas with low awareness levels and maintain high awareness in districts where it is already prevalent. Overall, improving awareness of the ovulatory cycle among tribal women is essential for enhancing their reproductive health and overall well-being. The government of India has not explicitly endorsed the use of knowledge of the ovulatory cycle (KOC) as a family planning method; it has implemented various strategies in recent years to address population control. One such initiative is the Prerna Strategy, launched by Jansankhya Sthirata Kosh (JSK) or National Population Stabilization Fund, aimed at promoting a higher age of marriage for girls, delaying the onset of the first childbirth, and encouraging spacing between subsequent childbirths to safeguard the health of young mothers and infants. Couples adopting this strategy are appropriately recognised, contributing to a shift in community mindsets.

In addition, the Santushti Strategy, implemented by JSK, involves collaborating with private-sector gynaecologists and vasectomy surgeons to perform sterilization operations through a Public Private Partnership mode. Private hospitals or nursing homes achieving a target of 10 or more operations are duly recognized and rewarded following the strategy.

## Limitations

The existing literature on knowledge of the ovulation cycle is notably sparse, offering limited coverage of various aspects of the ovulatory cycle. This field is relatively new and is increasingly attracting attention from researchers across multiple disciplines. First, our study is limited to cross-sectional data where we focus on quantitatively assessing the relationship between socio-economic factors and women’s knowledge of the ovulation cycle. Second, our study’s sample is limited to women aged 15 to 49 years who belong to Scheduled tribes. Third, the data we used is the response by women which self-reported in the nature of NFHS and may influenced by bias. Despite these constraints, our study offers a realistic portrayal of the state of knowledge about the ovulation cycle in India among tribal women. By bridging the gaps between the limited existing literature and our research findings, a clear message emerges, underscoring the urgent need for comprehensive adolescent programs aimed at enhancing knowledge of the ovulation cycle and reproductive health in India.

## Data Availability

All the data are open and can be accessed at https://dhsprogram.com. The shape file of spatial data was downloaded from the Spatial Data Repository– Boundaries, The Demographic and Health Surveys Program, ICF International, funded by the United States Agency for International Development (USAID). Available from https://spatialdata.dhsprogram.com.
